# B cells are capable of independently eliciting rapid reactivation of encephalitogenic CD4 T cells in a murine model of multiple sclerosis

**DOI:** 10.1371/journal.pone.0199694

**Published:** 2018-06-26

**Authors:** Chelsea R. Parker Harp, Angela S. Archambault, Julia Sim, Mark J. Shlomchik, John H. Russell, Gregory F. Wu

**Affiliations:** 1 Department of Neurology, Washington University School of Medicine, St. Louis, MO, United States of America; 2 Department of Developmental Biology, Washington University School of Medicine, St. Louis, MO, United States of America; 3 Department of Immunology, University of Pittsburgh, Pittsburgh, PA, United States of America; 4 Department of Pathology & Immunology, Washington University School of Medicine, St. Louis, MO, United States of America; Klinikum rechts der Isar der Technischen Universitat Munchen, GERMANY

## Abstract

Recent success with B cell depletion therapies has revitalized efforts to understand the pathogenic role of B cells in Multiple Sclerosis (MS). Using the adoptive transfer system of experimental autoimmune encephalomyelitis (EAE), a murine model of MS, we have previously shown that mice in which B cells are the only MHCII-expressing antigen presenting cell (APC) are susceptible to EAE. However, a reproducible delay in the day of onset of disease driven by exclusive B cell antigen presentation suggests that B cells require optimal conditions to function as APCs in EAE. In this study, we utilize an *in vivo* genetic system to conditionally and temporally regulate expression of MHCII to test the hypothesis that B cell APCs mediate attenuated and delayed neuroinflammatory T cell responses during EAE. Remarkably, induction of MHCII on B cells following the transfer of encephalitogenic CD4 T cells induced a rapid and robust form of EAE, while no change in the time to disease onset occurred for recipient mice in which MHCII is induced on a normal complement of APC subsets. Changes in CD4 T cell activation over time did not account for more rapid onset of EAE symptoms in this new B cell-mediated EAE model. Our system represents a novel model to study how the timing of pathogenic cognate interactions between lymphocytes facilitates the development of autoimmune attacks within the CNS.

## Introduction

Multiple sclerosis (MS) is a debilitating autoimmune disease of the central nervous system (CNS) with an unknown etiology despite being the subject of intense study for over a century [1]. MS is characterized by the chronologically and spatially distinct formation of lesions (“plaques”) comprised of cellular and humoral inflammation, demyelination, and axonal damage. Experimental autoimmune encephalomyelitis (EAE) is the main animal model for MS used to investigate the cellular mechanisms of disease as well as to develop new MS treatments [2, 3]. Early experiments with EAE identified the CD4 T cell as both necessary and sufficient for disease and prompted further investigation into the characteristics of MHCII+ antigen presenting cells (APCs) responsible for the regulation of CD4 T cell behavior during neuro-inflammation [4].

In MS, B cell depletion therapies (BCDTs) have recently been shown to be highly effective at amelioration of disease [5, 6]. BCDT reduces relapses and decreases inflammatory lesions [5, 7] but does not affect cerebral spinal fluid (CSF) levels of immunoglobulin nor deplete the long-lived antibody-secreting plasma cells from within the CSF or other tissues [8, 9]. Various approaches with BCDT in EAE also demonstrate that B cells can have an enormous influence on cognate encephalitogenic T cell pathogenicity and highlight the importance of antibody-independent B cell functions for the pathogenesis of CNS autoimmunity [10–12]. B cells are not highly phagocytic yet are very efficient at presenting antigens acquired via receptor-mediated endocytosis [13–15]. Although the target antigens for MS are unknown, recombinant B cell receptors (BCRs) derived from CSF-localized B cell clones exhibit specificity for myriad CNS components [16–19]. Through the process of linked recognition, a non-auto-reactive B cell could still present self-peptide antigens associated with internalized immune complexes to activate auto-reactive CD4 T cells [20]. Understanding how B cell-mediated antigen presentation influences neuro-inflammation and tolerance in MS could lead to potent and more specific immunomodulatory therapies.

Our previous work demonstrated that B cells are capable of serving as the only APC during passive EAE [15]. However, transgenic mice with elevated B cell specificity for MOG (IgH^MOG^ mice) crossed to mice expressing MHCII exclusively by CD19^+^ B cells (CD19-B^MHCII^ mice)–referred to as CD19-B^MHCII^xIgH^MOG^ mice because CD19^Cre^ drives MHCII expression on B cells–develop passive EAE with a statistically significant and reproducible delay in onset compared to WT mice [15]. Thus, B cells are capable of propagating auto-antigen-specific CNS demyelination on their own but may be limited in their efficiency as APCs. To explore the kinetics of B cell cognate interactions during EAE, B^MHCII^xIgH^MOG^ mouse were bred to a Tamoxifen (Tam)-inducible CD20^Tam-Cre^ murine reagent generated by Shlomchik and colleagues [21]. By carefully controlling of the timing of cognate interactions between B cells and CD4 T cells during EAE, we have observed a rapid induction of disease, demonstrating that B cells can efficiently drive auto-reactive CD4 T cell responses targeting the CNS.

## Materials & methods

### Mice

WT C57BL/6 (B6) mice, MOG-specific TCR-transgenic (2D2) mice, and UBC^Tam-Cre^ mice were obtained from The Jackson Laboratory (Bar Harbor, ME). CD19-B^MHCII^xIgH^MOG^ mice, in which B cells expressing a transgene driving a MOG-specific BCR exclusively express MHCII, were generated as previously reported [15]. CD20^Tam-Cre^ mice [21] were bred to IAß^b^stop^flox/flox^xIgH^MOG^ mice [15] to generate CD20-B^MHCII^xIgH^MOG^ and UBC^Tam-Cre^ mice were bred to IAß^b^stop^flox/flox^ mice to obtain UBC^MHCII^ mice [21, 22]. Littermates from IAß^b^stop^flox/flox^xIgH^MOG^ crossed to CD20^Tam-Cre^xIAß^b^stop^flox/flox^ breedings were used for comparison during experiments. 2D2 mice were crossed with CD19-B^MHCII^xIgH^MOG^ mice, which lack a complete endogenous CD4 T cell compartment. Three-week-old 2D2xCD19-B^MHCII^xIgH^MOG^ progeny received a thymic transplant from WT animals to support 2D2 thymocyte development as previously described, and were harvested after 8–12 weeks [23]. Animals were housed in a specific pathogen-free barrier facility at the Washington University School of Medicine (WUSM). All breeding and experimental protocols were performed in accordance with protocols reviewed and approved by the WUSM Animal Studies Committee/Institutional Animal Care and Use Committee (IACUC). Death was not considered an experimental outcome for any of the experiments reported herein. Nevertheless, humane endpoints were considered for all experiments, and according to IACUC protocols all experiments were designed to minimize suffering and distress of mice.

### EAE

Active EAE with rhMOG (Children’s Hospital of Pennsylvania) was induced by s.c. immunization of 150 μg rhMOG protein (residues 1–125 of human MOG protein [23]) emulsified in CFA (Sigma, St. Louis, MO). Mice received 200ng Pertussis toxin (Enzo, Farmingdale, NY) i.p. at the time of immunization and 48h later. Passive EAE was induced by adoptive transfer of 5x10^6^ MOG-specific Thy1.1+ or CD45.1+ encephalitogenic CD4 T cells, as previously reported [23]. Transfer experiments in which encephalitogenic donor T cells were ‘rested’ *in vivo* in an MHCII-deficient host were performed by initially transferring 5x10^6^ encephalitogenic CD4 T cells into MHCII-deficient IAß^b^stop^flox/flox^xIgH^MOG^ mice. Three weeks later, CD4 T cells were isolated from those mice and immediately to transferred into CD19-B^MHCII^xIgH^MOG^ or WT mice. In other experiments, MHCII expression was induced in UBC^MHCII^ and CD20-B^MHCII^xIgH^MOG^ mice by oral gavage with 5mg Tam in 50μL corn oil. Tam was either administered three days prior to donor T cell transfer or at various time points after donor T cell transfer. MHCII expression was verified by flow cytometric analysis of blood collected from the cheek. Mice were observed daily and clinical scores were determined by a five point scoring system, as follows: 0 = no disease; 1 = limp tail; 2 = mild hind limb paresis; 3 = severe hind limb paresis; 4 = complete hind limb plegia or quadriplegia; 5 = moribund or dead.

### Flow cytometry

Spleens were harvested from Avertin-anesthetized mice and single cell suspensions were treated with ACK erythrocyte lysis buffer. Mice were perfused with 25mL of ice-cold PBS and CNS tissues were isolated. Mononuclear cells were purified from homogenized brains and spinal cords by centrifugation for 30 min in 30% Percoll (GE Healthcare) solution as previously reported [22]. Cells were incubated with the anti-Fc receptor antibody 2.4G2 prior to the addition of staining antibodies. Intracellular cytokine (BD Cytofix/Cytoperm Plus, BD Biosciences, San Diego CA) and FoxP3 staining (Mouse Regulatory T cell Staining Kit #1, eBioscience San Diego, CA) of donor CD4 T cells was performed in accordance with kit protocols. The following anti-mouse antibodies were purchased from BD Biosciences (San Jose, CA): anti-CD69 FITC clone H1.2F3, anti-CD45 FITC, anti-GR-1 PE clone RB6-8C5, anti-CD3e PE-CF594 clone 145.2C11, anti-B220 PE-CF594 clone RA3-6B2, anti-CD44 PerCP/Cy5.5 clone IM7, anti-CD19 APC clone 1D3. The following antibodies were purchased from eBioscience (San Diego, CA): anti-CD45.1 FITC clone A20, anti-Foxp3 PE clone FJK-16s, rat anti-IgG2a isotype PE clone eBR2a, anti-CD25 APC clone PC61.5, anti-IFNγ APC clone XMG1.2. The following antibodies were purchased from BioLegend (San Diego, CA): anti-IL-17 FITC clone TC11-18H10.1, anti-CD4 clone GK1.5 in FITC and BV 421, anti-CD19 PE clone 1D3, anti-GM-CSF PE clone MP1-22E9, anti-CD49d clone R1-2 in PE and PE/Cy7, anti-Thy1.1 PerCP clone OX-7, anti-CD11b clone M1.7 in PerCP/Cy5.5 and PE/Cy7, anti-GR-1 PE/Cy7 clone RB6-8C5, anti-MHCII clone M5/114.15.2 in AF700 and Pacific Blue, anti-CD44 AF700 clone IM7, anti-CD8α APC/Cy7 clone 53.6.7, anti-CD45 AF700 clone 30-F11 in AF700, Pacific Blue, and BV 510. Cells were acquired on a Gallios flow cytometer (Beckman Coulter) and analyzed with FlowJo software version 8.5.2 (TreeStar, Ashland, OR) with doublets excluded.

### Histology and immunofluorescence

Mice were sacrificed and perfused with 25mL ice cold PBS followed by 20mL 4% paraformaldehyde (Sigma-Aldrich). Spinal cord tissue was removed from the vertebrae and then fixed in 4% paraformaldehyde for more than 12 hours, followed by dehydration in 30% sucrose for 48 hours. The tissue was embedded in optimal cutting temperature media (TissueTek, Torrence, CA) and cut at 8–10μm thick sections using a Leica CM1900 cryostat (Germany). The WUSM Developmental Biology Histology & Microscopy Core stained sections with Luxol Fast Blue to detect myelin. Slides were examined by light microscopy using a Nikon 90i motorized upright digital microscope with camera and Metamorph software (Molecular Devices). Sections were also stained to quantify myelinated white matter using goat anti-MOG (Invitrogen) with isotype control goat IgG2a (abcam) and secondary antibody donkey anti-goat Alexa-555 (Invitrogen). Slides were mounted with Fluoroshield Mounting Medium with DAPI (abcam). Slides were examined by immunofluorescent microscopy using a Nikon 90i motorized upright digital microscope with CoolSNAP ES^2^ camera (Photometrics). Inflammation score was recorded by blinded observer using the following scale system: 0 = normal, no inflammation; 1 = mild inflammation with a few small regions of increased cellularity; 2 = moderate inflammation with one large lesion or several smaller inflammatory foci; 3 = severe inflammation with dense parenchymal infiltration and many large lesions; 4 = massive inflammation in which most of the white matter has dense cellularity. Demyelination was quantified by blinded calculation of lesion area in thoracic spinal cord sections using Metamorph software (Molecular Devices, Inc).

### Statistical analysis

All data generated are reported as mean ± the standard error of the mean (SEM). Unpaired t-tests were used for comparison of cellular infiltrates. Time-to-EAE-onset incidence curves were compared by Log-rank (Mantel-Cox) tests. Group effects were compared via analysis of variance (ANOVA) with Tukey’s test or Kruskal-Wallis test with Dunn’s test for multiple comparisons when assumptions were not violated. Mann-Whitney tests with two-tailed P values were performed for flow cytometry assays quantifying lymphocytes in the CNS. All statistical analyses were completed using PRISM 7 software (GraphPad).

## Results

### B cell antigen presentation is insufficient to initiate spontaneous autoimmune CNS demyelination

Increasing the frequency of MOG-specific B cells in 2D2 mice, in which T cell receptors are highly specific for MOG, routinely leads to spontaneous inflammatory demyelination within the spinal cord and optic nerve, indicating that naïve MOG-specific B and T cells collaborate to induce neuro-inflammation [24, 25]. Additionally, transgenic SJL/J mice with MOG-specific T cells require an intact B cell compartment for susceptibility to spontaneous EAE [26]. To test the hypothesis that antigen presentation by B cells can independently trigger spontaneous EAE, we crossed CD19-B^MHCII^xIgH^MOG^ with 2D2 mice and salvaged 2D2 CD4 T cell development in progeny by thymus transplant. While both 2D2 and 2D2xIgH^MOG^ mice with WT MHCII expression spontaneously developed optic neuritis, only 2D2xIgH^MOG^ mice developed spontaneous EAE, confirming previously published data (Table 1). However, 2D2xCD19-B^MHCII^xIgH^MOG^ mice were completely resistant to both optic nerve inflammation and spontaneous EAE (Table 1) despite a substantial reconstitution of the CD4 T cell compartment with MOG-specific T cells as reported [23]. These results suggested that antigen-specific B cells alone were not sufficient to elicit CD4 T cell-dependent spontaneous inflammatory demyelination within the CNS, even when a high frequency of cognate, auto-reactive T cells were present. When these data are considered in conjunction with our previous observation that EAE is delayed in CD19-B^MHCII^xIgH^MOG^ mice [15], it suggests that B cells are intrinsically less efficient than other APCs in their ability to initiate and direct CD4 T cell auto-reactivity to MOG.

**Table 1:** B cells are not sufficient APCs to support spontaneous EAE or optic neuritis.

Genotype	Optic Neuritis	Thymus Transplant	Spontaneous EAE
2D2	5/11	No	0/11
2D2xIgH^MOG^	3/3	No	15/19
2D2xCD19-B^MHCII^xIgH^MOG^	0/19	Yes	0/21
WT hMOG*	3/5	No	5/5

* Active EAE (not spontaneous)

### Tamoxifen-inducible systems for induction of MHCII expression

**Fig 1:**
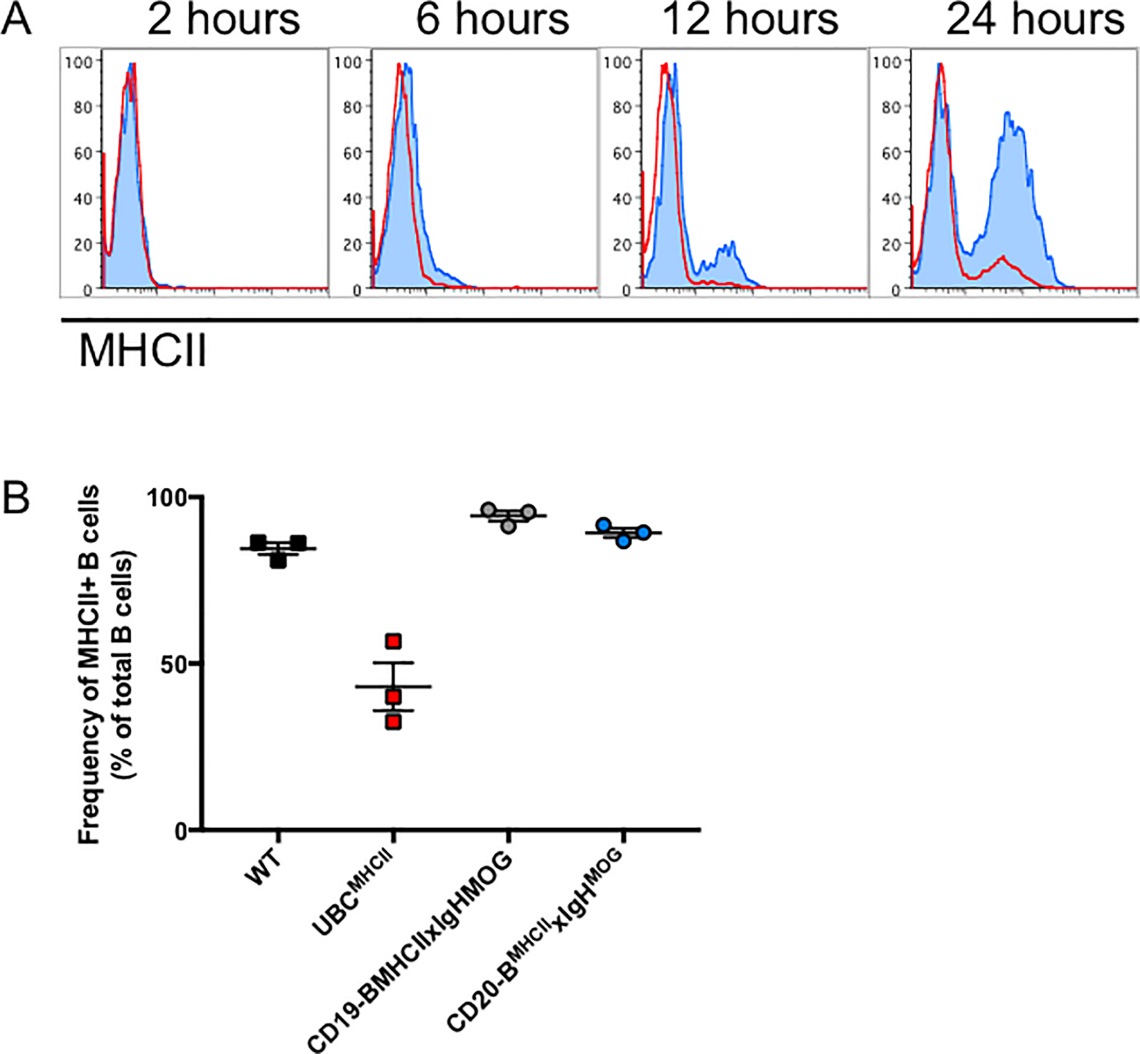
Temporal regulation of MHCII expression *in vivo*. (A) MHCII expression on peripheral blood B cells collected from UBC^MHCII^ (red line) and CD20-B^MHCII^xIgH^MOG^ (blue shaded) at several time points after Tam administration. Data are representative of two experiments, (n = 2 mice per time point from each genotype). (B) 72 hours post Tam treatment, spleens were harvested from naïve mice 72 hours after oral gavage with Tam for UBC^MHCII^ (red squares) and CD20-B^MHCII^xIgH^MOG^ (blue circles) or corn oil vehicle for WT (black squares) and CD19-B^MHCII^xIgH^MOG^ mice (grey circles). The frequency of MHCII+ B cells as a percentage of total B cells is presented as mean ± SEM from n = 3 mice per genotype.

By breeding the IAß^b^stop^flox/flox^xIgH^MOG^ mouse to Tam-inducible CD20^Tam-Cre^ mice, we aimed to temporally regulate MHCII expression exclusively by B cells. To assess the efficiency of recombination at the IA-ß locus following Tam administration, peripheral blood was collected from CD20-B^MHCII^xIgH^MOG^ and UBC^MHCII^ mice prior to oral gavage with Tam and at various time points thereafter. MHCII expression was undetectable prior to Tam treatment for both CD20-B^MHCII^xIgH^MOG^ and UBC^MHCII^ mice similar to the two hour timepoint (Fig 1A). 52.3 ± 4.7% (Mean ± SEM) of B cells from CD20-B^MHCII^xIgH^MOG^ mice expressed MHCII 24 hours after Tam administration (Fig 1A). After 72 hours, the mean frequency of MHCII+ B cells in CD20-B^MHCII^xIgH^MOG^ spleens (89.3 ± 1.4%) was similar to that of WT (84.5 ± 1.8%) and CD19-B^MHCII^xIgH^MOG^ mice (94.3 ± 1.5%) (Fig 1B). Following Tam administration, fewer B cells in UBC^MHCII^ mice expressed MHCII compared to WT B cells, yet MHCII was induced on other APC subsets in UBC^MHCII^ mice (Fig 1B).

### B cell-mediated EAE is delayed in CD20-B^MHCII^xIgH^MOG^ mice

**Fig 2:**
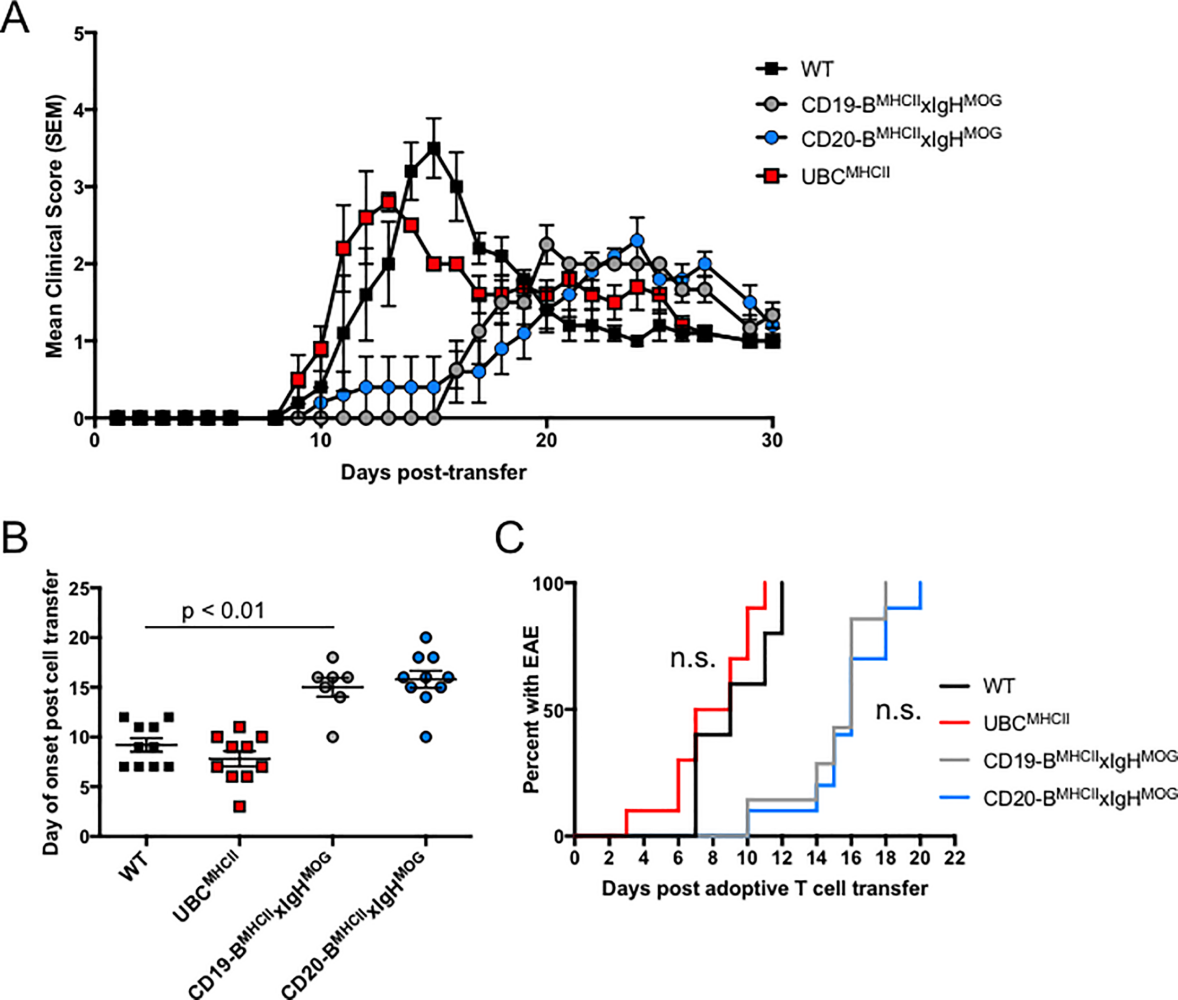
Tam-inducible MHCII expression models recapitulate WT and B-cell mediated adoptive transfer EAE models. (A) Mean ± SEM EAE scores recorded for CD20-B^MHCII^xIgH^MOG^ (blue circles), and UBC^MHCII^ (red squares) mice treated with Tam by oral gavage and WT (black squares), CD19-B^MHCII^xIgH^MOG^ (grey circles), treated with corn oil vehicle by oral gavage once, three days prior to receiving 5x10^6^ encephalitogenic CD4 T cells. Data is representative of three independent experiments with n = 3–5 mice per genotype. (B) Day of EAE onset post cell transfer for WT (black squares), UBC^MHCII^ (red squares), CD19-B^MHCII^xIgH^MOG^ (grey circles) treated with corn oil 72 hours prior to CD4 T cell transfer and CD20-B^MHCII^xIgH^MOG^ (blue circles) recipient mice treated with Tam 72 hours prior to CD4 T cell transfer. Graph shows mean day of onset ± SEM from two pooled, independent experiments with n = 2–4 mice per genotype. Unpaired t test performed to compare day of onset between WT and CD19-B^MHCII^xIgH^MOG^ mice. (C) Mice were treated with Tam 72 hours prior to CD4 T cell transfer. Time to EAE onset for WT (black) and UBC^MHCII^ (red) mice is not significantly different by log-rank test (p = 0.107). Time to EAE onset for CD19-B^MHCII^xIgH^MOG^ (grey), and CD20-B^MHCII^xIgH^MOG^ (blue) mice is not significantly different by log-rank test (p = 0.481). Incidence curves generated from two pooled, independent experiments with n = 2–4 mice per genotype.

Based on the high efficiency of MHCII induction by Tam administration, we hypothesized that expression of MHCII by B cells in CD20-B^MHCII^xIgH^MOG^ mice would manifest EAE similar to CD19-B^MHCII^xIgH^MOG^ mice. Thus, we treated CD20-B^MHCII^xIgH^MOG^ mice and UBC^MHCII^ mice with Tam three days prior to the adoptive transfer of encephalitogenic T cells. In WT mice, adoptive transfer of encephalitogenic CD4 T cells typically resulted in clinical and pathological signs within 10 days **(**Fig 2A**).** The time to onset and disease course for WT and UBC^MHCII^ mice treated with Tam prior to the adoptive transfer of CD4 T cells were not significantly different **(**Fig 2B and 2C**)**. As previously reported, CD19-B^MHCII^xIgH^MOG^ mice exhibited a statistically significant delay in the day of onset of EAE compared to WT mice, with EAE signs beginning on average at 15.0 ± 1.0 days following transfer of donor T cells [15]. CD20-B^MHCII^xIgH^MOG^ mice treated with Tam before T cell transfer exhibited similar disease kinetics, with an average day of EAE onset of 15.8 ± 0.9 days (Fig 2B and 2C). Hence, in our Tam-inducible MHCII system, B cell-restricted MHCII expression requires an extended period of time to facilitate disease mediated by encephalitogenic CD4 T cells compared to a full complement of MHCII+ APC subsets. These results indicate that the conditional and temporal regulation of MHCII *in vivo* is suitable for investigating the timing of B cell involvement during EAE.

### B cells are capable of inducing accelerated onset EAE

**Fig 3:**
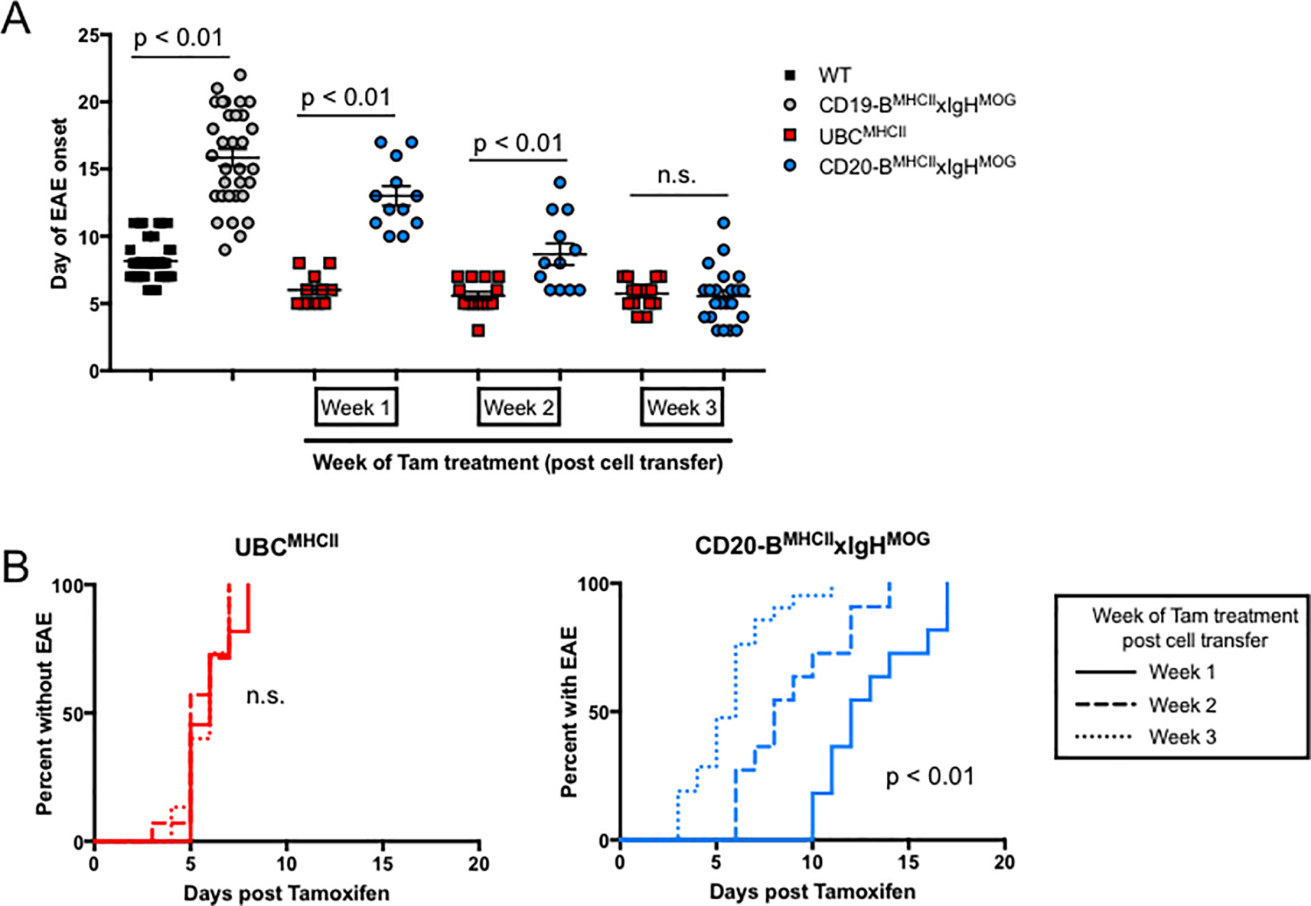
Tam treatment after T cell transfer results in accelerated B cell mediated EAE. (A) Mean ± SEM day of EAE onset for WT (black squares) and CD19-B^MHCII^xIgH^MOG^ (grey circles) post T cell transfer, and for UBC^MHCII^ (red squares) and CD20-B^MHCII^xIgH^MOG^ (blue circles) treated with Tam at either one week, two weeks, or three weeks post T cell transfer. Data is pooled from 12 different experiments with n = 1–5 mice per genotype and time point evaluated, p values calculated by unpaired t tests. (B) Time to EAE onset is not significantly different for UBC^MHCII^ mice (left graph) treated with Tam at either Week 1 (solid line), Week 2 (dashed line), or Week 3 (dotted line) post CD4 T cell transfer. Time to EAE onset is significantly delayed for CD20-B^MHCII^xIgH^MOG^ mice (right graph) treated with Tam at either Week 1 (solid line), compared to Week 2 (dashed line), or Week 3 (dotted line) post CD4 T cell transfer. Incidence curves generated from is pooled from 12 different experiments with n = 1–5 mice per genotype and time point, with significance evaluated by log-rank test.

Antigen acquisition and presentation by B cells is implicated as a critical precursor to CD4 T cell activation and demyelination in B cell-dependent models of EAE [27–29]. It is possible that B cells require more time to collect and process antigen because they are generally sequestered in peripheral lymphoid tissues where CNS antigens are less accessible. We reasoned that B cell-mediated EAE would be delayed regardless of when MHCII expression was induced relative to CD4 T cell transfer. To test this, encephalitogenic CD4 T cells were transferred into CD20-B^MHCII^xIgH^MOG^ mice and UBC^MHCII^ mice prior to the induction of MHCII expression. No animals developed EAE prior to Tam treatment. Regardless of the amount of time between CD4 T cell transfer and Tam-induced MHCII expression, the time to EAE onset for UBC^MHCII^ mice after Tam administration was not significantly different; the average day of EAE onset for UBC^MHCII^ mice treated with Tam one week after T cell transfer was 6.0 ± 0.4 days post Tam, whereas the average for mice treated with Tam after two or three weeks post T cell transfer was 5.6 ± 0.3 days and 5.7 ± 0.3 days post Tam, respectively (Fig 3A and 3B). Surprisingly, we observed a rapid onset of EAE signs for CD20-B^MHCII^xIgH^MOG^ mice that was dependent on the length of time between T cell transfer and MHCII induction (Fig 3A and 3B). The mean day of onset for CD20-B^MHCII^xIgH^MOG^ mice was significantly delayed compared to UBC^MHCII^ mice when Tam was administered at either week one or week two after T cell transfer (Fig 3A**)**. However, when mice received Tam three weeks after CD4 T cell transfer, the mean time to EAE onset for CD20-B^MHCII^xIgH^MOG^ and UBC^MHCII^ mice was not significantly different **(**Fig 3B**)**. These results demonstrate that B cell-mediated EAE has the capacity to develop more quickly than even the conventional WT passive EAE model. Thus, our Tam-inducible MHCII expression system provides a means to assess the kinetic differences in neuro-inflammation coordinated by antigen-specific B cells.

### Accelerated EAE is not due to immune cell trafficking to the CNS prior to Tam administration

**Fig 4:**
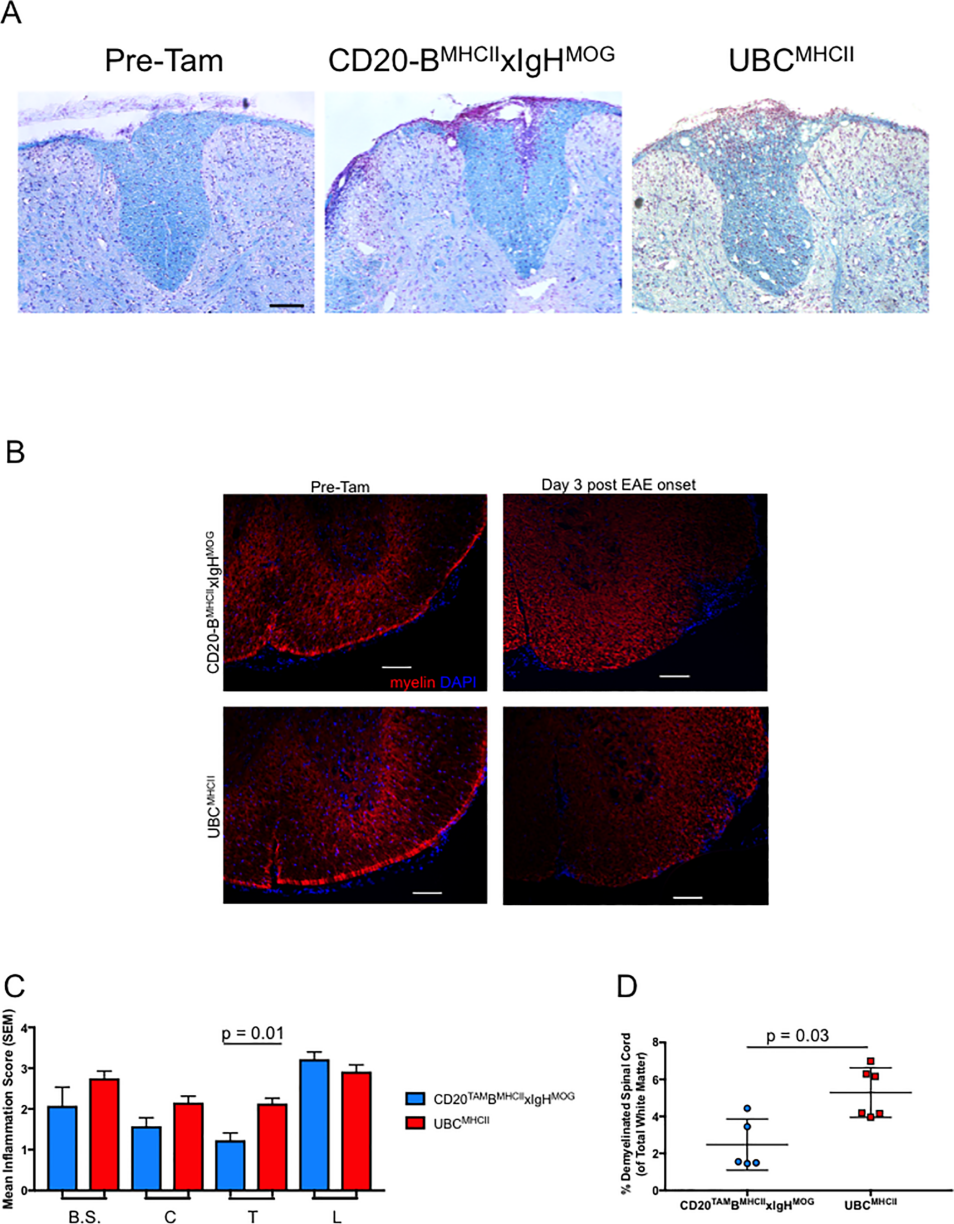
Inflammation and demyelination is not evident in spinal cords of encephalitic CD4 T cell recipients prior to Tam administration. (A) Representative spinal cord sections from recipients of encephalitogenic CD4 T cells (n = 5, CD20-B^MHCII^xIgH^MOG^ mice; n = 6, UBC^MHCII^ mice) were stained with Luxol Fast Blue, scale bar = 100um; all images generated from 10x magnification. (A) Spinal cords from CD20-B^MHCII^xIgH^MOG^ mice harvested three weeks after T cell transfer and before Tam administration (left). CD20-B^MHCII^xIgH^MOG^ mice treated with Tam three weeks after T cell transfer (middle) and harvested three days post EAE onset. UBC^MHCII^ mice treated with Tam three weeks after T cell transfer (right) and harvested three days post EAE onset. (B) Representative spinal cord sections from recipients of encephalitogenic CD4 T cells (at least mice 5 per genotype) were stained with antibodies to detect MOG, scale bar = 100um. Spinal cords from CD20-B^MHCII^xIgH^MOG^ mice (top left) and UBC^MHCII^ mice (bottom left) harvested three weeks after T cell transfer and before Tam administration. CD20-B^MHCII^xIgH^MOG^ (top right) and UBC^MHCII^ (bottom right) mice treated with Tam three weeks after T cell transfer and harvested three days post EAE onset. (C) Regions from rostral to caudal sections of spinal cords from CD20-B^MHCII^xIgH^MOG^ (blue) and UBC^MHCII^ mice (red) were harvested and scored for inflammation. Graph shows mean +/- SEM inflammation scores and Kruskal-Wallis test with Dunn’s correction for multiple comparisons was applied. B.S. = brainstem; C = cervical; T = thoracic; L = lumbar. (D) Mean (SD) percent area of demyelinated white matter was quantified for thoracic spinal cord sections from CD20-B^MHCII^xIgH^MOG^ (blue) and UBC^MHCII^ (red) mice. Significance determined by Mann-Whitney test with two-tailed p value.

**Fig 5:**
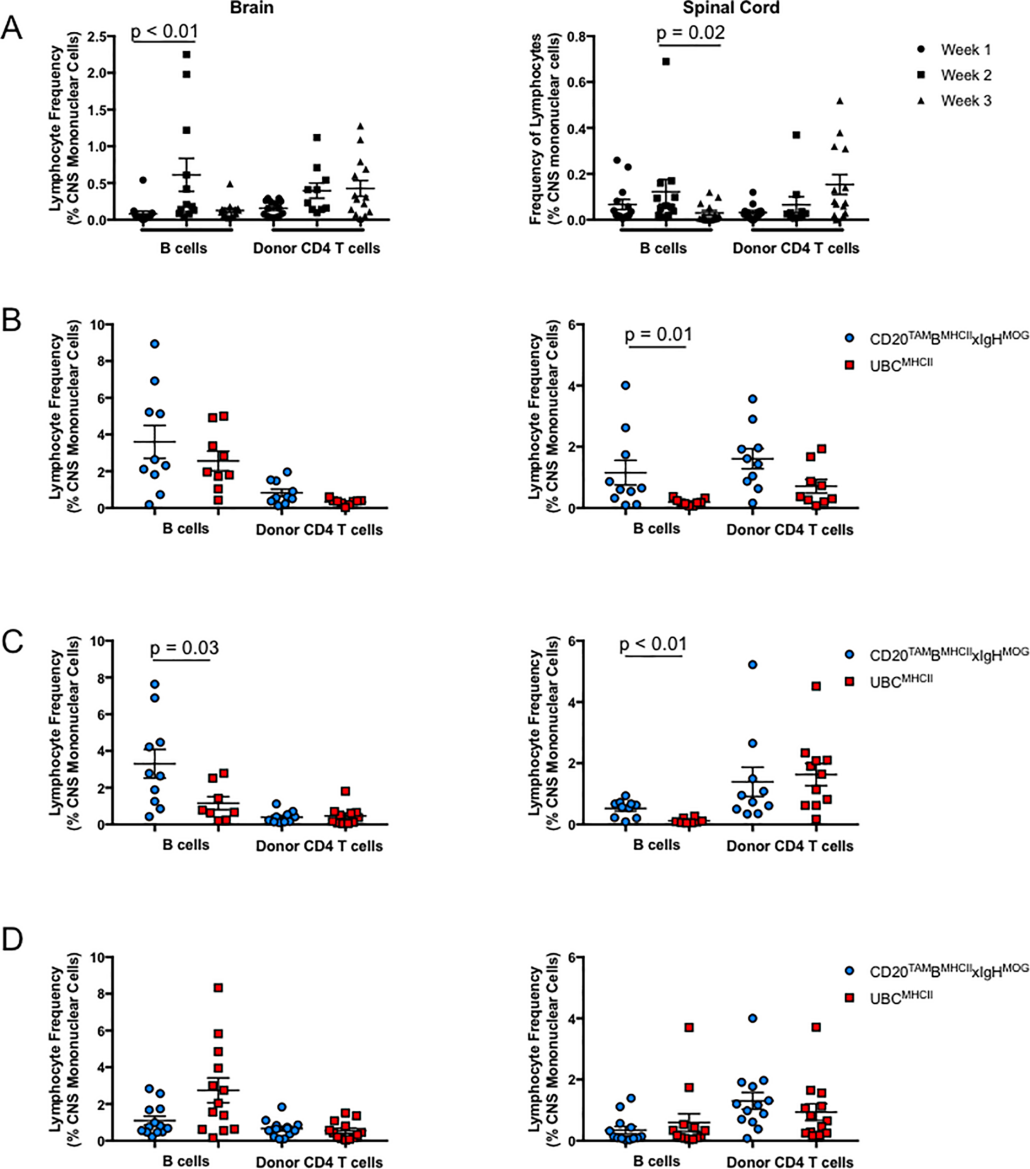
**Flow cytometric analysis of lymphocytes from brains (left) and spinal cords (right).** (A) Mean ± SEM frequency of B cells and donor CD4 T cells as a percent of total mononuclear cells in the CNS of mice at week 1 (circles), week 2 (squares), or week 3 (triangles) post CD4 T cell transfer. Data is pooled from CD20-B^MHCII^xIgH^MOG^ mice, UBC^MHCII^ mice, and IAß^b^stop^flox/flox^xIgH^MOG^ (Cre^-^) littermate controls from 9 different experiments with n = 3–5 mice at each time point prior to Tam treatment. Significance determined by Kruskal-Wallis test and Dunn’s correction for multiple comparisons. (B-D) Mean ± SEM frequency of B cells and donor CD4 T cells as a percent of total mononuclear cells in the brains (left) and spinal cords (right) harvested UBC^MHCII^ (red squares) and CD20-B^MHCII^xIgH^MOG^ (blue circles) mice approximately three days post EAE onset when mice were treated with Tam at (B) week 1, (C) week 2, or (D) week 3 post encephalitogenic CD4 T cell transfer. Data is pooled from 9 different experiments with n = 1–5 mice per genotype at each time point evaluated. Significance determined by Mann-Whitney test with two-tailed p value.

**Fig 6:**
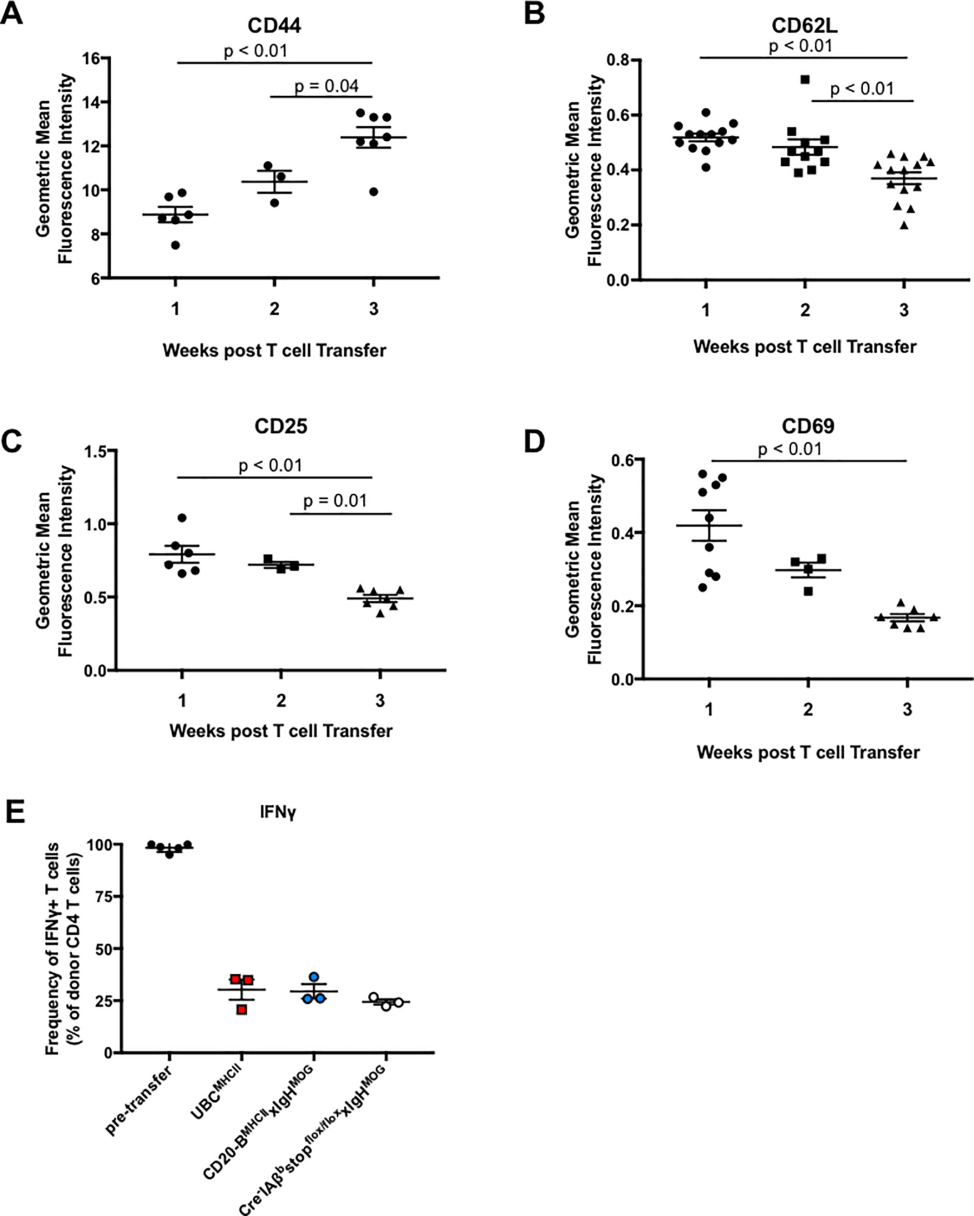
(A-D) Mean ± SEM of the geometric mean fluorescence intensity of activation marker expression on donor CD4 T cells harvested from spleens of recipient mice at week 1 (circles), week 2 (squares), or week 3 (triangles) post CD4 T cell transfer. Data is pooled from CD20-B^MHCII^xIgH^MOG^ mice, UBC^MHCII^ mice, and IAß^b^stop^flox/flox^xIgH^MOG^ (Cre^-^) littermate control recipients from five independent experiments with n = 2–4 mice per time point evaluated. Significance tested by one way ANOVA with Tukey’s test for multiple comparisons. Geometric mean fluorescence intensity of (A) CD44, (B) CD62L, (C) CD25, and (D) CD69 expression on donor CD4 T cells. (E) Frequency of IFNγ-expressing donor CD4 T cells pre-transfer and following *in vivo* incubation for three weeks in MHCII-deficient hosts. Graph is representative of n = 5 independent experiments with at least two mice from each genotype. Donor T cells were harvested from the spleen. Significance tested by Kruskal-Wallis test and Dunn’s correction for multiple comparisons.

A low frequency of CNS MHCII+ B cells can support neuro-inflammation in CD19-B^MHCII^xIgH^MOG^ mice with EAE [15]. Localization to the CNS compartment prior to Tam administration would give a small number of B cells ready access to both MOG antigens and cognate T cells primed to respond rapidly upon MHCII expression by the B cells. Hence, antigen-specific lymphocytes entering the CNS over time prior to the induction of MHCII expression could explain the increasingly rapid onset of EAE signs observed in CD20-B^MHCII^xIgH^MOG^ mice. To assess this possibility, the spinal cords of CD20-B^MHCII^xIgH^MOG^ mice and UBC^MHCII^ mice were examined for inflammation and demyelination before and after treatment with Tam three weeks post-T cell transfer. Histological examination of these tissues did not reveal any signs of inflammation or demyelination prior to Tam treatment (Fig 4A). Both CD20-B^MHCII^xIgH^MOG^ mice and UBC^MHCII^ mice that developed EAE after receiving Tam on week three post-T cell transfer had similar inflammatory foci (Fig 4A and 4B). Semi-quantitative assessment of inflammation of the spinal cord at the level of the brainstem and cervical, thoracic, and lumbar regions of the spinal cord revealed similar degrees of inflammation (Fig 4C). However, we observed a significant difference in the extent of inflammation between the genotypes at the thoracic level (Fig 4B). The difference in area of demyelinated white matter within the thoracic spinal cords of CD20-B^MHCII^xIgH^MOG^ and UBC^MHCII^ mice with EAE was significantly different, though this could be due to a higher mean EAE score for the UBC^MHCII^ mice (3.33 ± 0.4 vs. 2.4 ± 0.3) (Fig 4D). These observations were verified by flow cytometric analyses of the composition of infiltrating mononuclear cells in brain and spinal cord tissues examined prior to Tam treatment at various time points post T cell transfer (Fig 5 and S1 Fig). UBC^MHCII^, CD20-B^MHCII^xIgH^MOG^, and MHCII deficient Cre^-^IAß^b^stop^flox/flox^xIgH^MOG^ littermate control mice were harvested one, two, or three weeks post T cell transfer but prior to Tam treatment and brains and spinal cords were analyzed by flow cytometry to detect infiltrating B cells and donor CD4 T cells. Prior to Tam treatment, these recipient mice are all MHCII-deficient and analysis of donor T cell cytokine production after incubation in different genotypes does not indicate that the genotype of the recipient influences encephalitogenicity over time (Fig 6 and S2 Fig). While the mean frequency of B cells in the brain at week two post cell transfer was significantly different compared to week one post cell transfer (p < 0.01), there was no difference at week three and no overall increase in the frequency of lymphocytes in the CNS over time prior to Tam treatment (Fig 5A). Altogether, very few infiltrating lymphocytes were detected in the brain and spinal cord before MHCII expression was induced, and the cellular composition of CNS tissues from experimental mice was comparable to the number of B cells and endogenous CD4 T cells detected in the CNS of naïve WT mice (S1 Fig). Taken together this suggests that the rapid onset of disease is not a result of anticipatory migration of CD4 T or B cells into the CNS. Flow cytometry of CNS tissues harvested from CD20-B^MHCII^xIgH^MOG^ mice three days post-EAE onset showed that the frequency of B cells in the brains and spinal cords tended to be more variable when Tam is administered soon after T cell transfer (Fig 5B–5D**).** Additionally, although there is a higher frequency of B cells in the CNS of CD20-B^MHCII^xIgH^MOG^ mice with EAE when treated with Tam two weeks after T cell transfer (Fig 5C), the difference is not apparent when treated with Tam three weeks after T cell transfer (Fig 5D). These results suggest that the rapid onset of EAE signs observed in CD20-B^MHCII^xIgH^MOG^ mice treated with Tam weeks after T cell transfer is not the result of enhanced localization of B cells or T cells in the CNS compartment.

### Changes in donor T cells over time do not account for rapid onset of B cell-mediated EAE

It is possible that donor encephalitogenic CD4 T cells become more pathogenic over time prior to MHCII induction in CD20-B^MHCII^xIgH^MOG^ mice. To rule out the possible effects of increasing donor CD4 T cell activation, peripheral CD4 T cell markers were assayed by flow cytometry at various time points post-transfer and prior to Tam administration. The mean fluorescence intensity (MFI) of CD44, a marker of effector-memory CD4 T cells, increased over time while CD69, a marker of T cell activation, decreased over time (Fig 6A and 6D). The MFI of two other markers, CD62L, which is expressed by naïve T cells homing to secondary lymphoid tissues, and CD25, expressed by activated T cells and regulatory T cells, also decreased over time in MHCII deficient recipients prior to Tam administration (Fig 6B and 6C). Hence, deviations in activation status were observed in donor CD4 T cells after incubation in MHCII-deficient hosts. However, a mixture of changes, inconsistent with a selection of a singular population of activated encephalitogenic CD4 T cells, was observed after prolonged incubation in the absence of cognate interactions.

Subsequently, we reasoned that if the pre-Tam time period induces changes in donor T cells to enhance their encephalitogenic potential, we would then be able to induce rapid-onset EAE in WT or CD19-B^MHCII^xIgH^MOG^ mice by re-transferring the T cells into new naïve hosts. If the observed changes in donor T cells are responsible for the increasingly rapid EAE onset observed in CD20-B^MHCII^xIgH^MOG^ mice, donor T cells collected after an initial incubation in an MHCII-deficient host would induce rapid-onset EAE after a subsequent passive transfer into naïve recipients. Therefore, MHCII deficient Cre^-^IAß^b^stop^flox/flox^xIgH^MOG^ mice received encephalitogenic CD4 T cells, which were harvested from spleens and lymph nodes after three weeks of *in vivo* incubation. These freshly isolated T cells were MACS-purified by CD4 positive selection and immediately transferred into naïve WT or CD19-B^MHCII^xIgH^MOG^ recipients. The average day of onset was 18.2 for CD19-B^MHCII^xIgH^MOG^ receiving encephalitogenic CD4 T cells rested for three weeks in a MHCII-deficient environment, similar to the day of onset described previously for CD19-B^MHCII^xIgH^MOG^ mice receiving freshly *in vitro* generated donor CD4 T cell lines (Table 2**).** Likewise, the day of onset for WT and UBC^MHCII^ mice was similar to the day of onset for WT mice whether donor CD4 T cells were rested *in vivo* for three weeks or transferred immediately. Flow cytometry to detect intracellular cytokines revealed the frequency of IFNγ-producing donor T cells harvested three weeks after transfer into an MHCII-deficient recipient is drastically reduced compared to the frequency of cytokine-producing donor CD4 T cells prior to transfer. This same reduction in IFNγ production was observed in CD4 T cells harvested from CD20-B^MHCII^xIgH^MOG^ and UBC^MHCII^ mice prior to Tam administration (Fig 6E and S2 Fig). Taken together, the data indicate that prolonged incubation in a MHCII-deficient environment prior to Tam treatment does not lead to enhanced encephalitogenicity of donor CD4 T cells. Indeed, it is apparent that B cells are sufficient to provide the necessary APC functions to rapidly re-stimulate rested effector memory T cells to initiate EAE symptoms.

**Table 2:** Accelerated EAE is not induced by CD4 T cells harvested from MHCII-deficient hosts.

Recipient Genotype	Donor CD4 T cells	Incidence of EAE	Mean Day of onset (± SEM)
WT	*in vivo* incubated*	8/9	6.2 ± 0.6
CD19-B^MHCII^xIgH^MOG^	*in vivo* incubated	9/13	16.7 ± 2.3

* *≥* 3 weeks

## Discussion

The contributions of antigen specificity, antigen presentation, and cytokine production by individual APC populations on secondary T cell activation are complex and poorly understood. In MS and EAE, variation in T cell activation and effector lineage may be due to subtle differences in the abilities of APCs to acquire and present certain antigens at different times. For example, B6 mice are susceptible to MOG_35-55_ peptide-induced active EAE even if MHCII expression is restricted to DCs or if B cells are genetically ablated [23, 28, 30, 31]. However, resistance to MOG protein-induced active EAE can be seen in B cell deficient mice, mice with DC-specific MHCII expression, or mice in which B cells are MHCII deficient [28, 32, 33]. Taken together, it is apparent that B cell antigen presentation is critical for supporting CNS auto-reactivity to whole protein antigens.

Whether B cells facilitate initial CD4 T cell activation toward protein antigens in EAE has not been directly examined to date. In restricting MHCII expression to B cells, we found that the transgenic combination of B and T cell receptor specificities for MOG no longer evoked spontaneous inflammatory demyelination within the spinal cord (Table 1). While it is possible that 2D2xCD19-B^MHCII^xIgH^MOG^ mice do not develop spontaneous EAE due to the incomplete reconstitution of CD4 T cells by thymic grafting, our method has been utilized previously with success [23] and wild-type levels of CD4 T cells can be observed in secondary lymphoid tissue 9 weeks post-graft [23]. Other constraints such as a limited humoral MOG response in 2D2xCD19-B^MHCII^xIgH^MOG^ mice are unlikely to limit inflammatory demyelinating disease, as we believe that soluble MOG-specific antibody is not essential for EAE development. This is based on our observation that passive transfer of MOG-specific antibodies is not sufficient for disease development in CD19-B^MHCII^ mice [15] and the observation by the Zamvil group that mice in which MOG-specific antibody is tethered to B cells (and therefore incapable of being secreted) are susceptible to EAE [33]. While we did not collect serum from our cohort of 2D2xIgH^MOG^ mice, either with or without MHCII expression restricted to B cells, we have detected MOG-specific IgG in CD19-B^MHCII^xIgH^MOG^ mice, suggesting that antibody production in 2D2xCD19-B^MHCII^xIgH^MOG^ mice would not be absent or deficient. The lack of spontaneous EAE or optic neuritis in 2D2xCD19-B^MHCII^xIgH^MOG^ mice is in agreement with the previously reported limitations for B cells to prime CD4 T cells [22, 34]. On the other hand, B cells can indeed prime naïve T cells as long as valid B cell antigens are delivered to them [35]. Hence, in the EAE model, limitations in B cell APC function may be rooted in restricted access to relevant auto-antigens sequestered in the CNS. Hence, the responsibility for CD4 T cell activation and initiation of demyelination in spontaneous EAE most likely falls on DCs, which have been found to be capable of independently initiating spontaneous optic neuritis [23]. Whether the physical location of DCs in and around the CNS [36, 37] or the intrinsic ability for DCs to prime CD4 T cells [38] bestows this capacity for disease initiation is not apparent. Clearly, these features and others are not mutually exclusive.

Given the limitations for B cell antigen presentation in initiating disease, along with the restrictions in eliciting full recall production of cytokines by MOG-specific CD4 T cells in secondary responses [22], we sought to assess the limitations of B cells in supporting cognate CD4 T cell-mediated CNS autoimmunity after priming during EAE. Using a Tam-inducible MHCII expression system, we now show that antigen presentation by B cells is sufficient to induce EAE signs just as rapidly as EAE induced by a full complement of MHCII+ APCs. This result is contrary to our expectations given the reproducible delay in disease onset when B cells function as the sole APC during EAE [15]. Our findings raise questions regarding what drives accelerated disease onset in CD20-B^MHCII^xIgH^MOG^ mice. One critical component of neuro-inflammation worthy of consideration is lymphocyte trafficking. For example, the reduced variability in B cell frequency detected in the CNS of CD20-B^MHCII^xIgH^MOG^ mice with EAE induced by Tam administration after T cell transfer (Fig 5B–5D**)** could be explained by chronological changes in the concentration of chemotactic factors driving lymphocytes to the CNS. This theory could also explain the increasingly rapid onset of EAE symptoms observed for CD20-B^MHCII^xIgH^MOG^ mice treated with Tam after CD4 T cell transfer, as immune cells may traffic to, or organize within, the CNS more quickly if chemokines promoting B cell accumulation were up-regulated.

Our data support a model in which non-B cell APCs prime CD4 T cells toward myelin antigens, enabling subsequent cognate interactions with B cell APCs to support auto-reactive B cell proliferation and differentiation. Clinical studies have demonstrated that memory B cells and plasmablasts are the most common B cell subtype in the CSF of patients with MS [39]. This expansion of antigen-specific B cells during CNS autoimmunity could amplify cognate interactions between dysregulated B and CD4 T cells, which in turn could independently drive neuro-inflammation and relapses at later stages of MS. The high frequency of antigen-specific B cells in our EAE system may imitate the prevalence or expansion of B cells in MS patients [40–42]. During an MS remission, previously primed encephalitogenic T cells rest in the periphery (reflected in our EAE model as the period of time prior to Tam treatment). Of note, peripheral B cells from relapsing-remitting MS patients exhibit exaggerated pro-inflammatory cytokine responses that can directly promote T cell activation [43–45]. Increased MHCII expression promotes lymphocyte trafficking to the myelin-containing CNS compartment to initiate plaque formation (mimicked by what we observed soon after Tam treatment). It is possible that BCDTs eliminate pathogenic B cell antigen presentation, reducing germinal center reactions that may be occurring in the periphery and CNS. Studying the interdependency and amplification of pro-inflammatory interactions between dysregulated lymphocytes could lead to more targeted therapeutic interventions that specifically ablate the pathogenic effects of B cells *in situ*.

The reproducibly rapid onset of EAE that we have demonstrated shows that the time required for B cell trafficking from the periphery to the CNS is not a major contribution to the observed delay in disease onset for CD19-B^MHCII^xIgr mice. However, our findings raise questions about where antigen presentation needs to occur in the context of B cell-driven CNS autoimmunity. While germinal center reactions mainly occur in the spleen and lymph nodes, various reports describe formation of functional ectopic lymphoid follicles in the CNS of MS patients and in mice with EAE [46–48]. Exclusion of B cells from the CNS results in reduced severity of EAE induced by rhMOG yet exacerbates EAE induced by MOG_35-55_ peptide, indicating that access to the CNS is required for EAE mediated by B cell antigen presentation yet can also be important for regulatory B cells to ameliorate disease [49–51]. In contrast, Tesfagiorgis et al. found that antigen-nonspecific B cells are recruited to the CNS compartment while myelin-specific B cells remain in the peripheral draining lymph nodes in a model of active EAE that requires B cells [52]. In our model, the relative infrequency of B cells observed in the CNS of mice that develop EAE may reflect the ease with which MOG-specific B cells capture their soluble protein antigens to present to T cells within the CNS compartment. The formation of ectopic lymphoid follicles in the CNS could create an environment in which very few B cells have ready access to antigen and ample interactions with many cognate encephalitogenic CD4 T cells.

Overall, the results described herein reveal that myelin-specific B cells are not able to support spontaneous EAE, although they have the potential to rapidly induce EAE with similar kinetics as WT APCs. Our new inducible MHCII expression EAE system enables precise control of the timing of cognate interactions between B cells and CD4 T cells, revealing that B cells do not harbor an intrinsic deficit in the promotion of auto-reactive CD4 T cell responses targeting the CNS.

## Supporting information

S1 Fig Pre-tamoxifen immune cells do not accumulate within the spinal cord over time. Flow cytometric analysis was used to determine mean +/- SEM absolute number of B cells (A) and CD4 T cells (B) isolated from spinal cords (SC). CD20-B^MHCII^xIgH^MOG^ mice, UBC^MHCII^ mice, and Cre-IAß^b^stop^flox/flox^xIgH^MOG^ littermate controls harvested at week 1 (filled circles), week 2 (squares), or week 3 (triangles) post CD4 T cell transfer in comparison to lymphocytes in the spinal cords of naïve WT mice (open circles). Graphs show absolute number of donor CD4 T cells for mice harvested prior to Tam treatment and number of endogenous CD4 T cells from WT mice. Data is pooled from CD20-B^MHCII^xIgH^MOG^ mice, UBC^MHCII^ mice, and Cre- Cre-IAß^b^stop^flox/flox^xIgH^MOG^ littermate controls from 9 different experiments with n = 3–5 mice at each time point prior to Tam treatment. Significance determined by Kruskal-Wallis test with Dunn’s correction for multiple comparisons, with alpha = 0.05.
(TIFF)

S2 Fig Frequency of pro-inflammatory encephalitogenic T cells before and after transfer to MHCII-deficient hosts. Intracellular cytokine expression of IFNγ, IL-17, and GM-CSF by CD4 T cells prior to adoptive transfer is represented by black circles. After ‘resting’ in MHCII-deficient mice for 3 weeks, CD4 T cells were harvested from spleens of UBC^MHCII^ (red squares), CD20-B^MHCII^xIgH^MOG^ (blue circles) or CD20^Tam-Cre-^IAß^b^stop^flox/flox^xIgH^MOG^ (white circles) littermates and tested for intracellular cytokine expression. Kruskal-Wallis nonparametric test with Dunn’s correction for multiple comparisons did not identify significant differences in the percentage of T cells expressing various cytokines after incubation in MHCII-deficient mice with different genotypes (p>0.05).
(TIFF)
