# Supplementary figures and images for: B cells are capable of independently eliciting rapid reactivation of encephalitogenic CD4 T cells in a murine model of multiple sclerosis
Source: PLoS One. 2018 Jun 26;13(6):e0199694. doi: 10.1371/journal.pone.0199694 (PMC6019098; doi:10.1371/journal.pone.0199694)

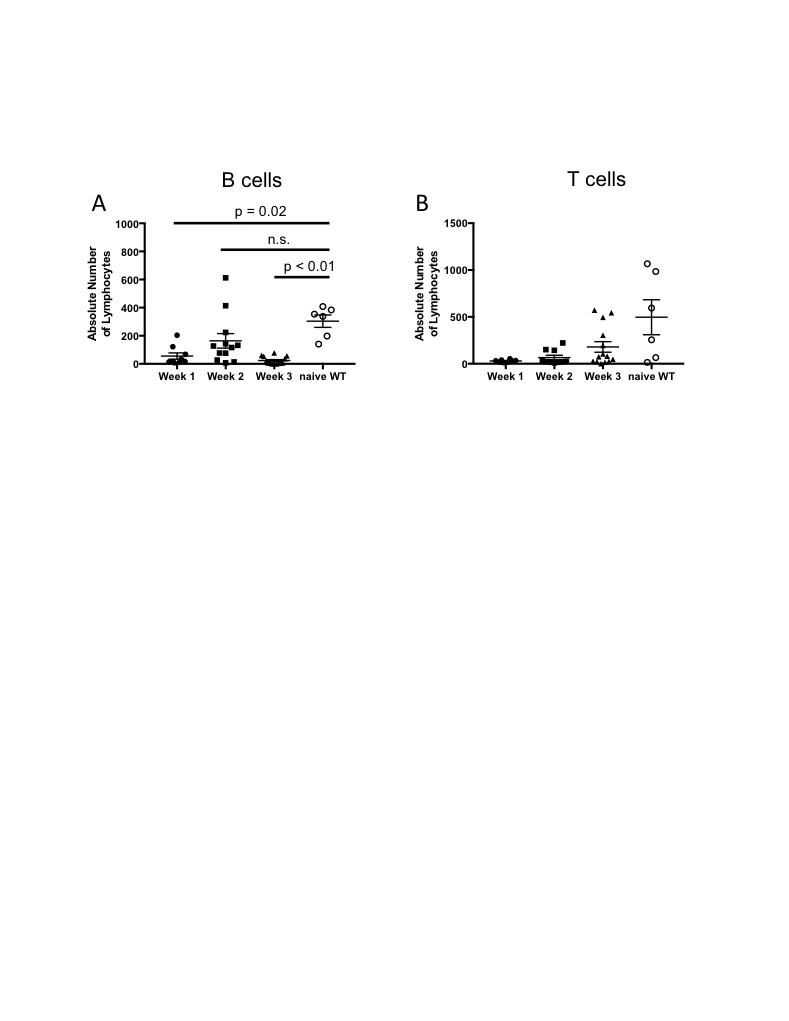

Supplement: S1 Fig — Flow cytometric analysis was used to determine mean +/- SEM absolute number of B cells (A) and CD4 T cells (B) isolated from spinal cords (SC). CD20-BMHCIIxIgHMOG mice, UBCMHCII mice, and Cre-IAßbstopflox/floxxIgHMOG littermate controls harvested at week 1 (filled circles), week 2 (squares), or week 3 (triangles) post CD4 T cell transfer in comparison to lymphocytes in the spinal cords of naïve WT mice (open circles). Graphs show absolute number of donor CD4 T cells for mice harvested prior to Tam treatment and number of endogenous CD4 T cells from WT mice. Data is pooled from CD20-BMHCIIxIgHMOG mice, UBCMHCII mice, and Cre- Cre-IAßbstopflox/floxxIgHMOG littermate controls from 9 different experiments with n = 3–5 mice at each time point prior to Tam treatment. Significance determined by Kruskal-Wallis test with Dunn’s correction for multiple comparisons, with alpha = 0.05. (TIFF) [file pone.0199694.s001.tiff]

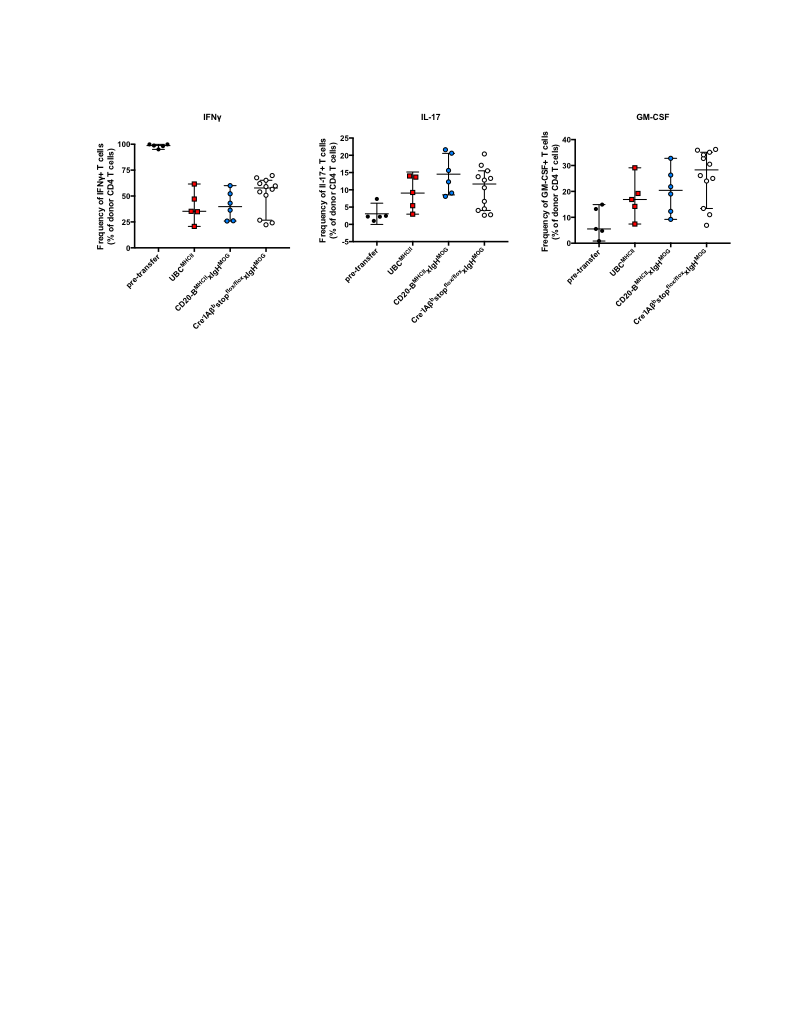

Supplement: S2 Fig — Intracellular cytokine expression of IFNγ, IL-17, and GM-CSF by CD4 T cells prior to adoptive transfer is represented by black circles. After ‘resting’ in MHCII-deficient mice for 3 weeks, CD4 T cells were harvested from spleens of UBCMHCII (red squares), CD20-BMHCIIxIgHMOG (blue circles) or CD20Tam-Cre-IAßbstopflox/floxxIgHMOG (white circles) littermates and tested for intracellular cytokine expression. Kruskal-Wallis nonparametric test with Dunn’s correction for multiple comparisons did not identify significant differences in the percentage of T cells expressing various cytokines after incubation in MHCII-deficient mice with different genotypes (p>0.05). (TIFF) [file pone.0199694.s002.tiff]
